# The effectiveness of 0.5 mg and 1mg of semaglutide in patients with type two diabetes and predictors of response: a retrospective cohort study

**DOI:** 10.3389/fendo.2024.1395651

**Published:** 2024-08-14

**Authors:** Sara Alenzi, Abdullah Alzahrani, Afnan Aljaloud, Kamayel Alanazi, Sumaiah J. Alarfaj

**Affiliations:** ^1^ Department of Pharmacy Practice, College of Pharmacy, Princess Nourah Bint Abdulrahman University, Riyadh, Saudi Arabia; ^2^ Department of Pharmaceutical Services, Security Forces Hospital Program, Riyadh, Saudi Arabia

**Keywords:** semaglutide, diabetes mellitus, obesity, glucagon-like peptide 1 analog, ozempic, Saudi

## Abstract

**Background:**

Semaglutide is a glucagon-like peptide-1 receptor agonists (GLP-1-RAs) approved for the treatment of type 2 diabetes mellitus (T2DM) at doses up to 1 mg. The results from randomized control trials and real-world studies revealed that weekly semaglutide was associated with significant improvements in HbA1c and body weight. To our knowledge, no study assessed the effectiveness of using semaglutide for patients with T2DM in the Saudi population. We aim to assess the effectiveness of once weekly SC 0.5 and 1 mg of semaglutide on HbA1c and weight reduction in patients with T2DM in the Saudi population within 12 months of use, evaluate the predictors of response, and compare the effect of the two doses.

**Method:**

This is a retrospective cohort study conducted at Security Force Hospital in Riyadh, Saudi Arabia. Using electronic medical records of patients with type two diabetes who received semaglutide 0.5 or 1 mg for a total duration of at least 12 months of use.

**Results:**

Within the study period of semaglutide use, HbA1c significantly decreased from baseline by -2.1% (-2.3 to -1.91, 95% CI) (P <0.001). While the mean change in weight was -6.19 kg (-6.66 to -5.72, 95% CI) (P<0.001). Moreover, BMI, FBG, total cholesterol, LDL, and TG all decreased significantly from baseline (p<0.001). When comparing the sub-groups of 0.5 and 1 mg doses, although results were numerically favorable of 1 mg, there were no statistically significant differences in HbA1c % (-2.1 ± 1.8 vs. -2.1 ± 1.9, p-value= 0.934, respectively), and weight (-6.1 ± 5 vs. -6.2 ± 4.4 kg, p-value=0.837, respectively). Significant predictors of HbA1c reduction were the duration of DM, baseline HbA1c, and insulin therapy. While the significant predictor for weight reduction was insulin therapy.

**Conclusion:**

This study is document the effectiveness of once-weekly SC semaglutide on glycemic control and weight loss in real-world practice. We recommend a starting goal dose of 0.5 mg and gradual increase of dose based individual patient response. further studies are needed to assess the effectiveness and tolerability of various semagltude doses.

## 1 Introduction

Diabetes mellitus (DM) is a diverse group of metabolic diseases characterized by chronically elevated blood glucose as their defining feature (1). In addition to hyperglycemia, DM is associated with metabolic abnormalities in metabolism and results in various micro and macrovascular complications (2). In Saudi Arabia, the pooled prevalence of type 2 DM (T2DM) is estimated at 16.4%, and the number of patients with diabetes is expected to double by 2030 (3, 4). Treatment strategies for T2DM include lifestyle modification and pharmacotherapy. The American Diabetes Association emphasizes weight management as a vital component in achieving glycemic control (5). Glucagon-like peptide 1 receptor agonists (GLP-1-RAs) have been recently added to the market and have significant effects in achieving glycemic control and decreasing weight in patients with T2DM (6). Additionally, guidelines recommend GLP-1-RAs with proven cardiovascular (CV) benefit as one of the preferred options for add-on therapy in patients with T2DM and established atherosclotic CV disease after metformin and lifestyle intervention (7). Semaglutide is a once-weekly injectable GLP-1-RAs formulation that was approved in 2018 for the treatment of T2DM at doses up to 1 mg (8) (Product Monograph OZEMPIC ^®^ semaglutide injection, n.d.). The clinical efficacy and safety of semaglutide have been studied in SUSTAIN clinical trials with the main outcome of assessing the efficacy in reducing Hemoglobin A1c % (HbA1c), weight change, and the safety profile of semaglutide in patients with T2DM (9). They found that semaglutide 0.5 and 1 mg were associated with significant reduction in HbA1c and weight, regardless to their use as add on with insulin or other antidiabetic agents.(9, 10, 11). In real world studies, the SURE program (The Semaglutide Unabated Real-World Effectiveness) comprised nine prospective observational real-world studies investigating once weekly semaglutide initiation in routine clinical practice in 10 countries: Canada (CA), Denmark/Sweden (DK/SE), France, Germany, Italy, Spain, The Netherlands, Switzerland (CH), and the United Kingdom (UK). A pooled analysis of data from four SURE studies included (CA, DK/SE, CH, and UK) showed a significant HbA1c reduction from baseline to end of the study by –1.1% and a significant body weight loss by –4.7 kg (10). Overall, the results from RCTs and real-world studies revealed that weekly semaglutide was associated with statistically significant and clinically relevant improvements in HbA1c and body weight in a wide range of adults with T2DM (11–15).

In clinical trials, both 0.5mg and 1mg of semaglutide have been assessed against placebo and other antidiabetic medications (16–21). The effect of both 0.5 and 1 mg was reported against placebo showing a slightly higher numerical advantage for 1mg. However, a direct comparison between effect of 0.5 and 1mg was not highlighted. Moreover, semaglutide drug approval studies did not include middle eastern population in the RCTs and real-world studies in this population are lacking. Therefore, we aimed to assess the effectiveness of 0.5 and 1 mg of semaglutide on HbA1C and weight reduction in patients with T2DM in the Saudi population within 12 months of use and compare effectiveness between the 2 doses.

## 2 Materials and methods

### 2.1 Study design, setting, and treatment with semaglutide

This is an observational retrospective cohort study conducted at Security Force Hospital in Riyadh, Saudi Arabia. This tertiary care hospital has a 532 bed capacity which provide inpatient and outpatient services and a diabetes care center. The treatment with subcutaneous (SC), once weekly dose of semaglutide was based on the recommended starting dose of 0.25mg, then increasing to reach a maintenance dose of either 0.5mg or 1 mg(Product Monograph OZEMPIC ^®^ semaglutide injection, n.d.).

### 2.2 Study population

Using electronic medical records, we extracted a list of all patients who received subcutaneous (SC) once weekly dose of semaglutide from 1 January 2021 to 31 December 2021. The total number was 4211 patients. We screened the generated list and excluded duplicates; the remaining patients were 3346. We included patients not well controlled on metformin since it was the first line treatment recommended by ADA at the time (22). Inclusion criteria include the following: Age between 18 and 65 years, had a BMI of > 30 and HbA1c > 8%, were treated with metformin and reached the maximum tolerated dose, had at least one weight-related condition including hypertension, high cholesterol, or ischemic heart disease (IHD), and were maintained on the same dose of semaglutide (0.5 or 1 mg) during the 6 and 12-month follow up period. We excluded patients who were treated with another GLP1 against and patients who discontinued the treatment during the 1-year follow-up period without a documented reason for discontinuation. To insure mutually exclusive groups, we also excluded patients who did not remain on the same dose of 0.5 or 1 mg of semaglutide during the 6 and 12 months points.

### 2.3 Sample size and data collection process

Using the Scalex SP calculator (21) and an estimated diabetes prevalence of 16.4% and the absolute precision of 5% in predicting the prevalence with 95% confidence and taking into account the probable loss/attrition of 10%, we calculated the required sample size to be 235 patients, with Expected 95% CI for this sample size was (11.4%, 21.4%). We aimed to include 350 patients. To reach our desired sample size, we wanted to stratify patients according to the month of starting semaglutide. We aimed for a minimum of 30 patients each month to reach the desired sample size of 350 patients. The selection from each strata was made randomly using a random number generator website. The data collected retrospectively included age (years), gender, duration of DM (years), weight (kg), BMI (kg/m^2^), weight-related comorbid conditions (including Dyslipidemia, Hypertension, IHD, Diabetic retinopathy, diabetic nephropathy), concomitant medications (Sulfonylurea, Biguanide, Glinide, Thiazolidinedione, SGLT2 inhibitor, DPP4 inhibitor, and Insulin therapy) HbA1c (%), fasting blood glucose (FBG) (mmol/L), and lipid profile (mmol/L) at baseline, six months, and 12 months.

### 2.4 Study outcomes

The primary outcomes was to assess semaglutide effect on HbA1c and weight within 12 months of use and identify the predictors of change. The secondary outcome was to compare the effectiveness of 0.5mg and 1mg doses of semaglutide.

### 2.5 Ethical considerations

The research committee in Security Forces Hospital approved the study (SFH) (H-01-R-069, Research Number 22-633-69). The data confidentiality was maintained, and the collected data through REDCap were unidentifiable.

### 2.6 Statistical analysis

Statistical analyses were performed using the IBM SPSS software version 28. Continuous variables were checked for normality using visual examination of histograms, Q-Q plots, and Shapiro-Wilk test; and were presented as mean ± standard deviation. Categorical variables are shown as frequencies and percentages. Continuous variables were compared using two-sample t-test or paired t-test, while categorical variables were compared using the Chi-squared or Fisher’s exact tests as appropriate. Pearson’s correlation analysis was used to evaluate the correlations between study variables and General Linear Model Repeated Measures ANOVA to study the longitudinal changes among different study timepoints. To examine the association between study variables and the outcome of HbA1c change at 12 months from baseline and weight change at 12 months, we conducted simple and multiple linear regression. After checking of linear regression assumptions, significant variables from simple linear regression were entered into multiple linear regression model. Interaction was examined by stratification and, if present, by the inclusion of an interaction term in the model to test for statistical significance. All reported P-values are two-sided and P-value <0.05 was considered statistically significant.

## 3 Results

### 3.1 The baseline characteristics

**Figure 1:**
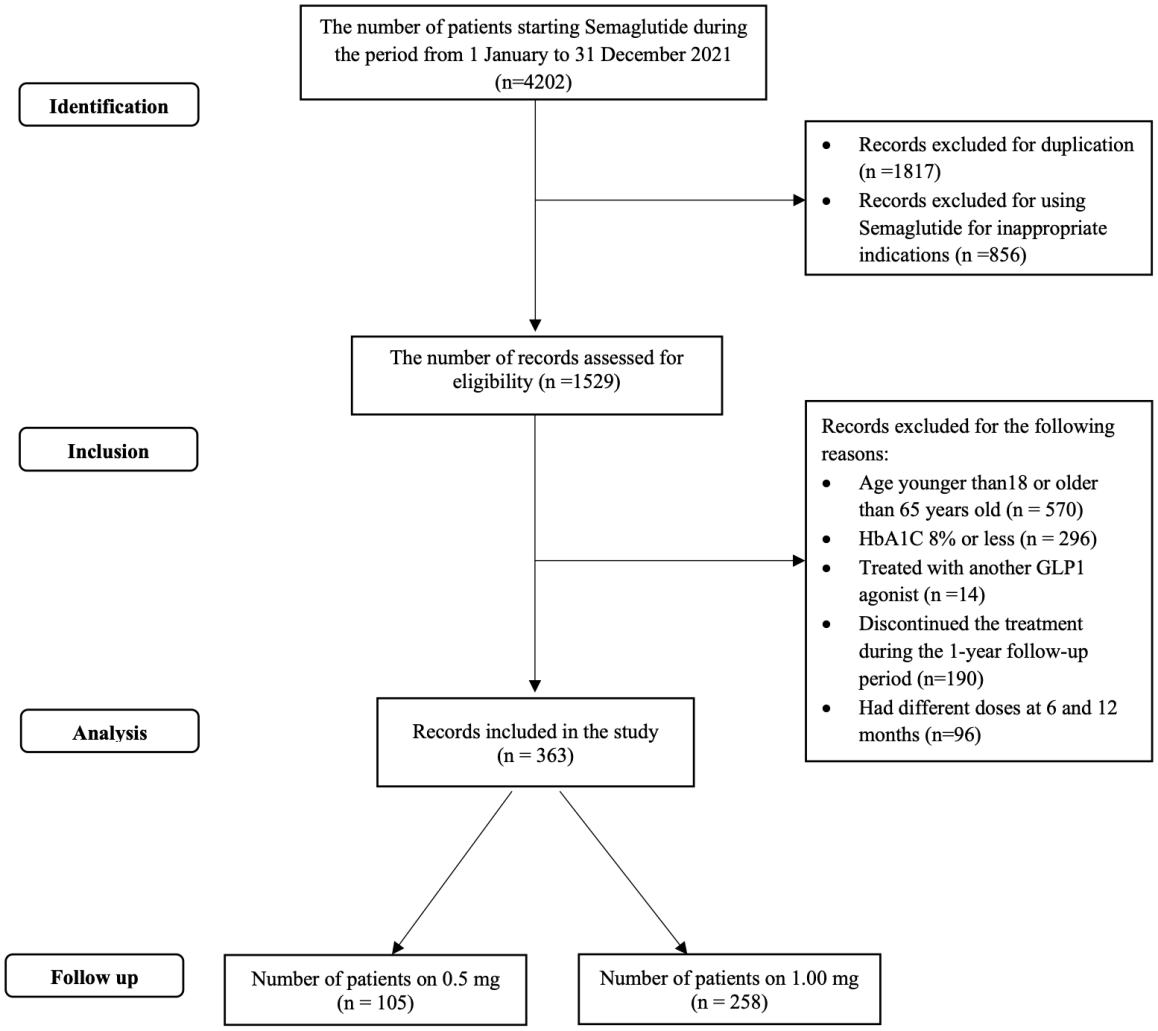
Included patients flow chart.

The total cohort of patients was 363. The process for patient selection and reasons for exclusion is depicted in **Figure 1**. The mean age of the patients was 52.6 ± 8.5 and the mean duration of diabetes was 14 years. Most of the patients had dyslipidemia, and about half of them had hypertension. A smaller percentage of patients had diabetic retinopathy and diabetic nephropathy, while 11% had ischemic heart disease. The most commonly used concomitant medication was biguanide, followed by SGLT2 inhibitors, and about half of the patients were on insulin therapy. The baseline HbA1c% was high (9.9% ± 1.5), and the baseline FBG was also elevated (11.5 mmol/L ± 3.7). The patients had a mean body weight of 94 kg ± 15.7 and a mean BMI of 36.2 kg/m^2^ ± 5.7. In this study, only 3 out of the 363 patients had missing data points in BMI, while one patient had missing data in cholesterol. Given the small number of missing values relative to the overall sample size, and since the missing data were not related to the study outcomes, no imputation techniques were applied. Further details are in **Table 1**.

**Table 1:** Baseline characteristics.

	Total	0.5 mg	1 mg	P value
Number of patients, n (%)	363	105 (28.9%)	258 (71.1%)	
Age (years)	52.6 ± 8.5	53.8 ± 8.4	52.1 ± 8.5	0.092
Diabetes duration (years)	14.2 ± 7.8	13.8 ± 7.2	14.3 ± 8.1	0.603
Gender				
Male	180 (49.6%)	47 (44.8%)	133 (51.6%)	0.249
Female	183 (50.4%)	58 (55.2%)	125 (48.4%)	
Comorbidities				
Dyslipidemia	329 (90.6%)	90 (85.7%)	239 (92.6%)	0.048
Hypertension	192 (52.9%)	61 (58.1%)	131 (50.8%)	0.246
IHD	40 (11%)	18 (17.1%)	22 (8.5%)	0.025
Diabetic retinopathy	29 (8%)	8 (7.6%)	21 (8.1%)	1
Diabetic nephropathy	38 (10.5%)	7 (6.7%)	31 (12%)	0.185
Concomitant medications				
Sulfonylurea	97 (26.7%)	34 (32.4%)	63 (24.4%)	0.150
Biguanide	356 (98.1%)	104 (99%)	252 (97.7%)	0.461
Glinide	2 (0.6%)	0 (0%)	2 (0.8%)	0.588
Thiazolidinedione	5 (1.4%)	0 (0%)	5 (1.9%)	0.327
SGLT2 inhibitor	173 (47.7%)	47 (44.8%)	126 (48.8%)	0.490
DPP4 inhibitor	7 (1.9%)	2 (1.9%)	5 (1.9%)	1
Insulin therapy	183 (50.4%)	46 (43.8%)	137 (53.1%)	0.132
SU/insulin/TZD^a^	307 (84.6%)	95 (90.5%)	212 (82.2%)	0.054
Body weight (kg)	94 ± 15.7	94.3 ± 15.4	93.9 ± 15.9	0.801
BMI (kg/m^2^)	36.2 ± 5.7	36.7 ± 6	36 ± 5.6	0.335
HbA1c (%)	9.9 ± 1.5	9.8 ± 1.4	9.9 ± 1.5	0.355
FBG (mmol/L)	11.5 ± 3.7	11.1 ± 3.6	11.7 ± 3.8	0.184
Total cholesterol (mmol/L)	4.5 ± 1.2	4.2 ± 1.3	4.6 ± 1.1	0.015
HDL (mmol/L)	1.2 ± 0.3	1.2 ± 0.3	1.1 ± 0.3	0.316
LDL (mmol/L)	2.9 ± 1	2.7 ± 1.1	3 ± 1	0.004
Triglycerides (mmol/L)	1.9 ± 1.2	1.9 ± 1.6	2 ± 1	0.409

a Patient is taking either any insulin, SU, or TZD.

IHD, Ischemic Heart disease; SGLT2, Sodium-Glucose cotransporter 2; DPP4, Dipeptidyl Peptidase-4; SU, Sulfonylurea; TZD, Thiazolidinedione; BMI, Body Mass Index; HbA1c%, Glycated Hemoglobin; FBG, Fasting Blood Glucose; HDL, High-Density Lipoprotein; LDL, Low-Density Lipoprotein.

### 3.2 Overall reduction in HbA1C and weight outcome

Within 6 and 12 months of semaglutide use, the decrease in HbA1c from baseline was significant (95% CI) at 6 months was -1.48% (-1.66 to -1.31) (P <0.001), and at 12 months of use was -2.1 (-2.3 to -1.91) (P <0.001). While the mean change in weight was -3.36 kg (-3.7 to -3.03) (P<0.001) at 6 months and -6.19 (-6.66 to -5.72) (P<0.001) at 12 months. Moreover, BMI, FBG, total cholesterol, LDL, and TG all decreased significantly from baseline (p<0.001). Further details are in **Table 2**.

**Table 2:** Change in metabolic profile from baselines at 6 months and 12 months of use.

Variables	6 months	P value	12 months	P value
HbA1c (%)	-1.48 (-1.66 to -1.31)	<0.001	-2.1 (-2.3 to -1.91)	<0.001
FBG (mmol/L)	-2.13 (-2.57 to -1.69)	<0.001	-3.39 (-3.83 to -2.95)	<0.001
Body weight (kg)	-3.36 (-3.7 to -3.03)	<0.001	-6.19 (-6.66 to -5.72)	<0.001
BMI (kg/m^2^)	-1.29 (-1.42 to -1.16)	<0.001	-2.39 (-2.57 to -2.21)	<0.001
Total cholesterol (mmol/L)	-0.14 (-0.25 to -0.04)	0.01	-0.24 (-0.35 to -0.12)	<0.001
Triglycerides (mmol/L)	-0.19 (-0.29 to -0.08)	0.001	-0.26 (-0.35 to -0.17)	<0.001
HDL (mmol/L)	0 (-0.02 to 0.02)	0.889	0.01 (-0.02 to 0.03)	0.640
LDL (mmol/L)	-0.11 (-0.2 to -0.01)	0.033	-0.19 (-0.3 to -0.09)	<0.001

BMI, Body Mass Index; HbA1c%, Glycated Hemoglobin; FBG, Fasting Blood Glucose; HDL, High-Density Lipoprotein; LDL, Low-Density Lipoprotein.

### 3.3 Comparison of 0.5 and 1 mg semaglutide doses

**Figure 2:**
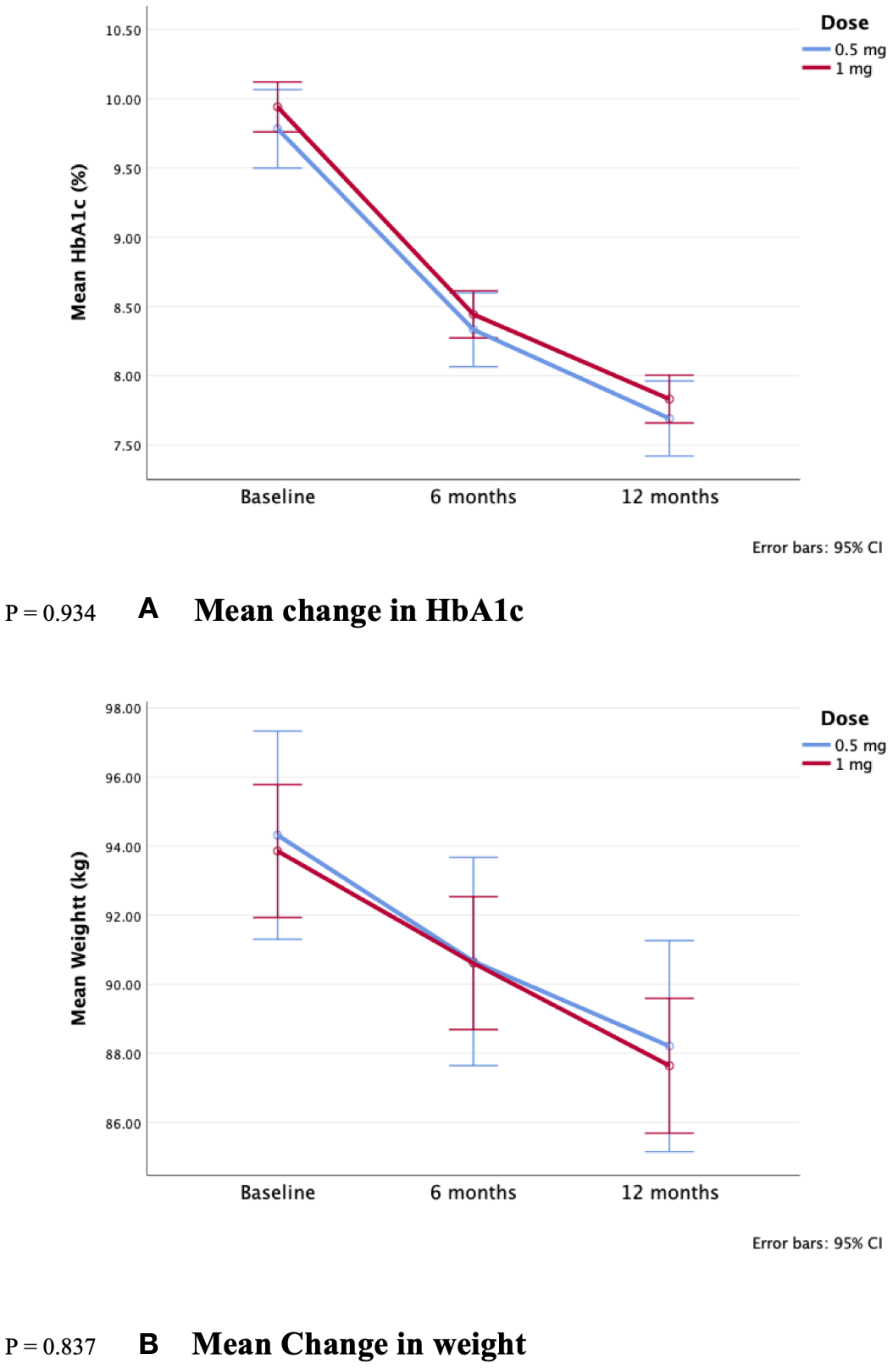
Comparison of 0.5 and 1 mg semaglutide use over 12 months reduction in **(A)** HbA1c and **(B)** Weight. P = 0.934. **(A)** Mean change in HbA1c. P = 0.837. **(B)** Mean Change in weight.

When comparing the sub-groups of 0.5 and 1 mg doses, although results were numerically favorable of 1 mg, there were no statistically significant differences in HbA1c (-2.1 ± 1.8 vs. -2.1 ± 1.9%, p-value= 0.934, respectively), and weight (-6.1 ± 5 vs. -6.2 ± 4.4 kg, p-value=0.837, respectively) (**Figure 2**). Additionally, all other changes were not statistically significant (p > 0.05). Further details are in **Table 3**; **Figure 2**.

**Table 3:** Sub-group comparison of semaglutide 0.5 vs. 1 mg effect on metabolic profile within 12 months .

Variable change at 12 months	0.5 mg group ± SD	1 mg group ± SD	P value
Weight	-6.1 ± 5	-6.2 ± 4.4	0.837
BMI	-2.4 ± 2	-2.4 ± 1.6	0.902
HbA1c	-2.1 ± 1.8	-2.1 ± 1.9	0.934
FBG	-3.4 ± 4.2	-3.4 ± 4.2	0.998
Cholesterol	-0.1 ± 1.1	-0.3 ± 1.1	0.235
HDL	-0.1 ± 1	0 ± 0.6	0.532
LDL	-0.1 ± 1	-0.2 ± 1	0.247
TG	-0.2 ± 1	-0.3 ± 0.9	0.746

BMI, Body Mass Index; HbA1c%, Glycated Hemoglobin; FBG, Fasting Blood Glucose; HDL, High-Density Lipoprotein; LDL, Low-Density Lipoprotein.

### 3.4 Predictors of effectiveness of semaglutide

#### 3.4.1 Predictor of change in HbA1c

We conducted univariate linear regression to assess the association between study variables and the change in HbA1c after 12 months. We found that age (B = 0.048; 95% CI 0.03 to 0.07; P<0.001), duration of DM (B = 0.065; 95% CI 0.04 to 0.09; P<0.001), cholesterol baseline (B=-0.259; 95% CI-0.42 to -0.1; P=0.002), HbA1c baseline (B = -0.834; 95% CI -0.93 to -0.74; P<0.001), FBG baseline(B= -0.143; 95% CI -0.19 to -0.09; P<0.001) and insulin use (B = 0.932; 95% CI 0.54 to 1.33; P<0.001) were significantly associated with HbA1c change (**Table S1**: in **Supplementary Files**). To adjust for confounding, all significant variables from univariate analysis were included in the multiple linear regression. Only the duration of DM, baseline HbA1c, and insulin therapy were significant predictors of HbA1c change. Further details are in **Table 4**.

**Table 4:** Multivariate analysis for HbA1c change.

	B	SE	BETA	p	CI
(Constant)	4.065	0.713	0	<0.001	2.66 to 5.47
Age	0.017	0.009	0.077	0.072	0 to 0.04
Duration of DM	0.024	0.01	0.102	0.018	0 to 0.04
Hypertension	0.099	0.145	0.027	0.498	-0.19 to 0.39
Cholesterol baseline	0.02	0.06	0.013	0.734	-0.1 to 0.14
HbA1c baseline	-0.82	0.047	-0.652	<0.001	-0.91 to -0.73
Any Insulin	0.866	0.147	0.221	<0.001	0.58 to 1.15

#### 3.4.2 Predictor of change in weight reduction

We conducted univariate linear regression to assess the association between study variables and the change in weight after 12 months. We found that only any insulin use (B = 1.278; 95% CI 0.29 to 2.27; P<0.011) was significantly associated with weight change (**Table S2**: in **Supplementary Files**). Since we only found 1 predictor of weight change, we were not able to do a multivariate analysis.

## 4 Discussion

Within our cohort, the average age was 52.6 ± 8.5 years, the average duration of DM was 14.2 ± 7.8 years, and the average baseline HbA1c was 9.9 ± 1.5%. The baseline characteristics between the 2 groups were comparable, except for higher number of patients with dyslipidemia and established IHD in the 1mg dose group. This indicates that clinicians might have a preference to use a higher dose of semaglutide for patients with increased CV risk, despite the lack of evidence supporting higher dose of semaglutide for risk reduction of major CV outcomes (7, 23).

Our results revealed that within 12 months, patients treated with semaglutide had a significant mean reduction in HbA1c (-2.1%) and FBG (-3.4mmol/L ± 4.2), weight was -6.19 kg, and BMI (-2.4 kg/m^2^± 1.8) from baseline. This was mostly comparable to results reported in previous studies. The mean reduction in HbA1c seems higher than in previous International studies ranging between 1.4% -1.8% for 0.5 and 1 mg (9, 12–14, 24). However, this higher reduction in HbA1c is consistent with another real-world study on Saudi population (25). This higher reduction might be attributed to the higher baseline HbA1c seen in our population, or due to sociodemographic difference. Further studies in Saudi patients are needed to explore these results.

Surprisingly, despite comparable baseline characteristics between the 0.5 and 1mg doses, the effectiveness between the two doses was comparable. The SUSTAIN RCT series have consistently used parallel group of 0.5 and 1mg of semaglutide to compare the effect of semaglutide against placebo and other antidiabetic medications, although the numerical reduction seemed higher in the higher dose, a direct comparison and statistical significance between the two doses was not provided. Additionally, we could not find a real-world study that compared the effectiveness between these 2 doses.

This study did not assess the safety and tolerability of semaglutide, primarily because of the retrospective nature of the study. As patients might discontinue the drug without reporting side effects. Additionally, we wanted to capture the true effect of different doses, so we excluded patient who discontinued the drug within 1 year. Therefore, comparison of side effect and adherence between 0.5 and 1 mg doses was not possible in this study. However, data from RCTs show an increase in some side effects with increasing the dose; like sever hypoglycemia and gastrointestinal side effects, which could affect adherence to semaglutide and limit long term benefit (20, 21, 26). Additionally, real-world studies assessing tolerability repot a lack of adherence of GLP-1RA possibly due to side effects (27, 28), which warrant further precaution when choosing to increase the goal dose.

The SUSTAIN FORTE trial compared the effect of semaglutide 2 mg vs 1mg in a similar patient population (patients on metformin and HbA1c >8%), and they found a significant reduction in both HbA1c and weight in the 2 mg doses as compared to the 1 mg dose. Results from our study support the SUSTAIN FORTE recommendation of gradual dose increase based on patient tolerability and outcome. Further real-world studies are needed to compare the efficacy and tolerabilty between different doses of 0.5, 1, and 2 mg studies Moreover, further investigation is warranted to determine who might benefit from higher doses and when higher doses are recommended.

Additionally, we identified baseline HbA1c to have a direct relation to reduction in HbA1c. this makes sense because a higher the baseline will result in a higher % reduction. Moreover, we found that glycemic control is inversely related to duration of DM diagnosis and insulin therapy, while weight loss is inversely related to insulin use.

Predictors of glycemic control have been costately reported to include the duration of DM diagnosis (29). It might be counterintuitive at first to think that insulin therapy is inversely related to glycemic control in patients using semagltide. However, Semaglutide uses multiple mechanisms to achieve glycemic including stimulating insulin secretion to levels of healthy patients, limiting glucagon secretion, and delaying gastric emptying which increase fullness, decrease food intake, and result in weight reduction. A possible explanation is that patients using external insulin might already have higher insulin resistance limiting the effectiveness of semaglutide in those patients. Moreover, insulin use is known to increase patients’ weight, which in term limit the extent to which semagltide have on both weight and glycemic control. Previous studies indicate a linear relationship between weight and HbA1c reduction, where every 1 kg reduction in weight correlates with a 0.1% reduction in HbA1c (30). Therefore, it’s not surprising that insulin use is a predictor for semaglutide effectiveness in achieving both glycemic and weight control.

We only found one observational study that investigated predictors of response to semaglutide in patients with T2DM. The study by Marzullo (14) confirms the effect of HbA1c baselines and duration of DM on predicting a reduction in HbA1c. Additionally, previous studies investigating predictors of response in Dulaglutide and Liraglutide indicate similar findings (31, 32). Furthermore, we found a statistically significant reduction in FBG, BMI, total cholesterol, and LDL in line with existing literature (15, 33).

Results from this study validate the results from previous studies of the significant improvement in glycemic and weight-loss benefit. Additionally, it adds to the existing literature a confirmation of improved reduction in HbA1c observed in Saudi patients who were not tested in RCTs. Moreover, this study was the first to provide a direct comparison and statistical significance assessment between the 0.5 and 1 mg doses of semaglutide and showed no statistical significance. Further real-world studies are needed to assess the needs, benefits, and down side of increasing the dose of semaglutide.

This study is limited by the inherent biases of retrospective design and being in a single center. Data were collected from medical records; so the quality of the data is not optimal. Additionally, several factors that might affect the outcome were not measured including adherence and lifestyle modifications.

## 5 Conclusion

Semaglutide once weekly SC injections were found to provide significant glycemic and weight loss benefits in patients with T2DM. We recommend a gradual dose increase based on patient response to dose. Starting with a goal dose of 0.5 mg and assessing patient achievement of benefit, then increase the dose as needed based on individual patient response. Further studies are needed to assess the effectiveness and tolerability of various semagltude doses.

## Data Availability

The data analyzed in this study is subject to the following licenses/restrictions: data was collected by the investigators in accordance with IRB approval. we have no consent to share the dataset publicly. Requests to access these datasets should be directed to IRB of security forces hospital and the primary/corresponding author. sjalarfaj@pnu.edu.sa.

## References

[1] Kharroubi AT. Diabetes mellitus: The epidemic of the century. World J Diabetes. (2015) 6:850. doi: 10.4239/wjd.v6.i6.850 26131326 PMC4478580

[2] David MN. Long-term complications of diabetes mellitus. New Engl J Med. (1993). doi: 10.1056/NEJM199306103282306 8487827

[3] Shaw JE Sicree RA Zimmet PZ. Global estimates of the prevalence of diabetes for 2010 and 2030. Diabetes Res Clin Pract. (2010) 87:4–14. doi: 10.1016/j.diabres.2009.10.007 19896746

[4] Jarrar M Abusalah MH Albaker W Al-Bsheish M Alsyouf A Al-Mugheed K. Prevalence of type 2 diabetes mellitus in the general population of Saudi Arabia 2000–2020: A systematic review and meta-analysis of observational studies. Saudi J Med Med Sci. (2023) 11:1. doi: 10.4103/sjmms.sjmms_394_22 36909010 PMC9997860

[5] Elsayed NA Aleppo G Aroda VR Bannuru RR Brown FM Bruemmer D. 9. Pharmacologic approaches to glycemic treatment: standards of care in diabetes—2023. Diabetes Care. (2023) 46:S140–57. doi: 10.2337/dc23-S009 PMC9810476 36507650

[6] Hinnen D. Glucagon-like peptide 1 receptor agonists for type 2 diabetes. Diabetes Spectr. (2017) 30:202–10. doi: 10.2337/ds16-0026 PMC5556578 28848315

[7] The American Diabetes Association (ADA). 9. Pharmacologic approaches to glycemic treatment: standards of care in diabetes—2024. Diabetes Care. (2024) 47:S158–78. doi: 10.2337/dc24-S009 PMC10725810 38078590

[8] Novo Nordisk Canada. OZEMPIC® (semaglutide)[package insert]. U.S. Food and Drug Administration website. https://www.accessdata.fda.gov/drugsatfda_docs/label/2017/209637lbl.pdf. Revised December 2017. Accessed July 2024.

[9] Andreadis P Karagiannis T Malandris K Avgerinos I Liakos A Manolopoulos A. Semaglutide for type 2 diabetes mellitus: A systematic review and meta-analysis. Diabetes Obes Metab. (2018) 20:2255–63. doi: 10.1111/dom.13361 29756388

[10] Rajamand Ekberg N Bodholdt U Catarig AM Catrina SB Grau K Holmberg CN. Real-world use of once-weekly semaglutide in patients with type 2 diabetes: Results from the SURE Denmark/Sweden multicentre, prospective, observational study. Prim Care Diabetes. (2021) 15:871–8. doi: 10.1016/j.pcd.2021.06.008 34183269

[11] Shi FH Li H Cui M Zhang ZL Gu ZC Liu XY. Efficacy and safety of once-weekly semaglutide for the treatment of type 2 diabetes: A systematic review and meta-analysis of randomized controlled trials. Front Pharmacol. (2018) 9:576. doi: 10.3389/fphar.2018.00576 29915538 PMC5994433

[12] Brown RE Bech PG Aronson R. Semaglutide once weekly in people with type 2 diabetes: Real-world analysis of the Canadian LMC diabetes registry (SPARE study). Diabetes Obes Metab. (2020) 22:2013–20. doi: 10.1111/dom.14117 PMC7689820 32538541

[13] Hansen KB Svendstrup M Lund A Knop FK Vilsbøll T Vestergaard H. Once-weekly subcutaneous semaglutide treatment for persons with type 2 diabetes: Real-world data from a diabetes out-patient clinic. Diabetic Med. (2021) 38:1–9. doi: 10.1111/dme.14655 34291491

[14] Marzullo P Daffara T Mele C Zavattaro M Ferrero A Caputo M. Real-world evaluation of weekly subcutaneous treatment with semaglutide in a cohort of Italian diabetic patients. J Endocrinol Invest. (2022), 1587–98. doi: 10.1007/s40618-022-01799-2 PMC9270295 35429298

[15] Berra CC Rossi MC Mirani M Ceccarelli Ceccarelli D Romano C Sassi L. Real world effectiveness of subcutaneous semaglutide in type 2 diabetes: A retrospective, cohort study (Sema-MiDiab01). Front Endocrinol (Lausanne). (2023) 13:1099451. doi: 10.3389/fendo.2022.1099451 36743930 PMC9889982

[16] Ahrén B Masmiquel L Kumar H Sargin M Karsbøl JD Jacobsen SH. Efficacy and safety of once-weekly semaglutide versus once-daily sitagliptin as an add-on to metformin, thiazolidinediones, or both, in patients with type 2 diabetes (SUSTAIN 2): a 56-week, double-blind, phase 3a, randomised trial. Lancet Diabetes Endocrinol. (2017) 5:341–54. doi: 10.1016/S2213-8587(17)30092-X 28385659

[17] Aroda VR Bain SC Cariou B Piletič M Rose L Axelsen M. Efficacy and safety of once-weekly semaglutide versus once-daily insulin glargine as add-on to metformin (with or without sulfonylureas) in insulin-naive patients with type 2 diabetes (SUSTAIN 4): a randomised, open-label, parallel-group, multicentre, multinational, phase 3a trial. Lancet Diabetes Endocrinol. (2017) 5:355–66. doi: 10.1016/S2213-8587(17)30085-2 28344112

[18] Sorli C Harashima S-I Tsoukas GM Unger J Karsbøl JD Hansen T. Efficacy and safety of once-weekly semaglutide monotherapy versus placebo in patients with type 2 diabetes (SUSTAIN 1): a double-blind, randomised, placebo-controlled, parallel-group, multinational, multicentre phase 3a trial. Lancet Diabetes Endocrinol. (2017) 5:251–60. doi: 10.1016/S2213-8587(17)30013-X 28110911

[19] Efficacy and safety of once-Weekly semaglutide versus exenatide ER in subjects with type 2 diabetes (SUSTAIN 3): A 56-Week, open-Label, randomized clinical trial. In: Diabetes Care. American Diabetes Association Inc (2018). p. 258–66. doi: 10.2337/dc17-0417 29246950

[20] Pratley RE Aroda VR Lingvay I Lüdemann J Andreassen C Navarria A. Semaglutide versus dulaglutide once weekly in patients with type 2 diabetes (SUSTAIN 7): a randomised, open-label, phase 3b trial. Lancet Diabetes Endocrinol. (2018) 6:275–86. doi: 10.1016/S2213-8587(18)30024-X 29397376

[21] Rodbard HW Lingvay I Reed J de la Rosa R Rose L Sugimoto D. Semaglutide added to basal insulin in type 2 diabetes (SUSTAIN 5): A randomized, controlled trial. J Clin Endocrinol Metab. (2018) 103:2291–301. doi: 10.1210/jc.2018-00070 PMC5991220 29688502

[22] American Diabetes Association. Pharmacologic approaches to glycemic treatment: Standards of medical care in diabetesd2021. Diabetes Care. (2021) 44:S111–24. doi: 10.2337/dc21-S009 33298420

[23] Marso SP Bain SC Consoli A Eliaschewitz FG Jódar E Leiter LA. Semaglutide and cardiovascular outcomes in patients with type 2 diabetes. New Engl J Med. (2016) 375:1834–44. doi: 10.1056/NEJMoa1607141 27633186

[24] Shi Q Nong K Vandvik PO Guyatt GH Schnell O Rydén L. Benefits and harms of drug treatment for type 2 diabetes: systematic review and network meta-analysis of randomised controlled trials. BMJ. (2023) 381:e074068. doi: 10.1136/bmj-2022-074068 37024129 PMC10077111

[25] Alsheikh A Alshehri A Alzahrani S Jammah AA Alqahtani F Alotaibi M. Evaluating the clinical effectiveness and safety of semaglutide in individuals with uncontrolled type 2 diabetes. Real-world evidence from Saudi Arabia: the observational, multicenter, 15-month EVOLUTION study. Diabetes Ther. (2024) 15:473–85. doi: 10.1007/s13300-023-01516-z PMC10838866 38110660

[26] O’Neil PM Birkenfeld AL McGowan B Mosenzon O Pedersen SD Wharton S. Efficacy and safety of semaglutide compared with liraglutide and placebo for weight loss in patients with obesity: a randomised, double-blind, placebo and active controlled, dose-ranging, phase 2 trial. Lancet. (2018) 392:637–49. doi: 10.1016/S0140-6736(18)31773-2 30122305

[27] Tofé S Argüelles I Mena E Serra G Codina M Urgeles JR. Real-world GLP-1 RA therapy in type 2 diabetes: A long-term effectiveness observational study. Endocrinol Diabetes Metab. (2019) 2(1):e00051. doi: 10.1002/edm2.51 30815578 PMC6354754

[28] Alarfaj SJ Ibrahim A Alshahrani J Alnuwaysir M Almutairi A Alwahhabi B. Effectiveness, tolerability, and pattern of liraglutide treatment use for weight loss: a mixed-methods cohort study. J Advanced Pharm Educ Res. (2022) 12:63–70. doi: 10.51847/WAv0CPT0BT

[29] Bitew ZW Alemu A Jember DA Tadesse E Getaneh FB Sied A. Prevalence of glycemic control and factors associated with poor glycemic control: A systematic review and meta-analysis. Inq (United States). (2023) 60:00469580231155716. doi: 10.1177/00469580231155716 PMC10071101 36852627

[30] Gummesson A Nyman E Knutsson M Karpefors M. Effect of weight reduction on glycated haemoglobin in weight loss trials in patients with type 2 diabetes. Diabetes Obes Metab. (2017) 19:1295–305. doi: 10.1111/dom.12971 28417575

[31] Toyoda M Yokoyama H Abe K Nakamura S Suzuki D. Predictors of response to liraglutide in Japanese type 2 diabetes. Diabetes Res Clin Pract. (2014) 106:451–7. doi: 10.1016/j.diabres.2014.09.052 25458335

[32] Berra CC Resi V Mirani M Folini L Rossi A Solerte SB. Clinical efficacy and predictors of response to dulaglutide in type-2 diabetes. Pharmacol Res. (2020) 159:104996. doi: 10.1016/j.phrs.2020.104996 32574827

[33] Di Folco U Vallecorsa N Nardone MR Pantano AL Tubili C. Effects of semaglutide on cardiovascular risk factors and eating behaviors in type 2 diabetes. Acta Diabetologica. (2022) 59(10):1287–94. 10.1007/s00592-022-01936-6 PMC9288662 35842847

