# Supplementary material for: The effectiveness of 0.5 mg and 1mg of semaglutide in patients with type two diabetes and predictors of response: a retrospective cohort study
Source: Front Endocrinol (Lausanne). 2024 Aug 14;15:1395651. doi: 10.3389/fendo.2024.1395651 (PMC11349510; doi:10.3389/fendo.2024.1395651)
Supplement: Supplementary file 1 [file Table1.docx]

# Suplemental file

Table S1: Univariate analysis for HbA1c change.

|  | **B** | **SE** | **BETA** | **p** | **CI** |
| --- | --- | --- | --- | --- | --- |
| Age | 0.048 | 0.011 | 0.22 | <0.001 | 0.03 to 0.07 |
| Female vs. male | -0.006 | 0.195 | -0.002 | 0.975 | -0.39 to 0.38 |
| Duration of DM | 0.065 | 0.012 | 0.273 | <0.001 | 0.04 to 0.09 |
| BMI baseline | 0.009 | 0.017 | 0.027 | 0.612 | -0.03 to 0.04 |
| Hypertension | 0.403 | 0.194 | 0.109 | 0.038 | 0.02 to 0.79 |
| Dyslipidemia | -0.092 | 0.334 | -0.015 | 0.783 | -0.75 to 0.57 |
| Cholesterol baseline | -0.259 | 0.081 | -0.166 | 0.002 | -0.42 to -0.1 |
| TG baseline | -0.1 | 0.083 | -0.063 | 0.233 | -0.26 to 0.06 |
| Retinopathy | 0.349 | 0.359 | 0.051 | 0.331 | -0.36 to 1.06 |
| Nephropathy | 0.548 | 0.317 | 0.091 | 0.085 | -0.08 to 1.17 |
| HbA1c baseline | -0.834 | 0.049 | -0.664 | <0.001 | -0.93 to -0.74 |
| FBS baseline | -0.143 | 0.025 | -0.289 | <0.001 | -0.19 to -0.09 |
| eGFR baseline | -0.002 | 0.002 | -0.05 | 0.348 | -0.01 to 0 |
| LDL baseline | -0.262 | 0.092 | -0.147 | 0.005 | -0.44 to -0.08 |
| HDL baseline | -0.6 | 0.333 | -0.094 | 0.072 | -1.26 to 0.06 |
| IHD | 0.432 | 0.31 | 0.073 | 0.165 | -0.18 to 1.04 |
| Sulfonylurea | -0.191 | 0.22 | -0.046 | 0.385 | -0.62 to 0.24 |
| Biguanide | -0.805 | 0.707 | -0.06 | 0.256 | -2.2 to 0.59 |
| Thiazolidinedione | 0.896 | 0.835 | 0.056 | 0.284 | -0.75 to 2.54 |
| SGLT2 | 0.288 | 0.195 | 0.078 | 0.139 | -0.09 to 0.67 |
| DPP4 | -0.171 | 0.709 | -0.013 | 0.81 | -1.56 to 1.22 |
| Insulin Use | 0.932 | 0.2 | 0.238 | <0.001 | 0.54 to 1.33 |

Table S2: Univariate analysis for weight change

|  | **B** | **SE** | **BETA** | **p** | **CI** |
| --- | --- | --- | --- | --- | --- |
| Age | 0.025 | 0.028 | 0.047 | 0.373 | -0.03 to 0.08 |
| Female vs. male | 0.329 | 0.479 | 0.036 | 0.492 | -0.61 to 1.27 |
| Duration of DM | -0.006 | 0.031 | -0.01 | 0.85 | -0.07 to 0.06 |
| BMI baseline | -0.036 | 0.042 | -0.044 | 0.4 | -0.12 to 0.05 |
| Hypertension | -0.384 | 0.479 | -0.042 | 0.424 | -1.33 to 0.56 |
| Dyslipidemia | 0.131 | 0.822 | 0.008 | 0.873 | -1.49 to 1.75 |
| Cholesterol baseline | -0.005 | 0.203 | -0.001 | 0.98 | -0.4 to 0.39 |
| TG baseline | 0.147 | 0.205 | 0.038 | 0.476 | -0.26 to 0.55 |
| Retinopathy | -1.04 | 0.882 | -0.062 | 0.239 | -2.77 to 0.69 |
| Nephropathy | -0.669 | 0.782 | -0.045 | 0.393 | -2.21 to 0.87 |
| HbA1c baseline | 0.046 | 0.163 | 0.015 | 0.779 | -0.27 to 0.37 |
| FBS baseline | 0.121 | 0.064 | 0.1 | 0.058 | 0 to 0.25 |
| ~~eGFR baseline~~ | ~~0.001~~ | ~~0.006~~ | ~~0.006~~ | ~~0.905~~ | ~~-0.01 to 0.01~~ |
| LDL baseline | -0.071 | 0.23 | -0.016 | 0.756 | -0.52 to 0.38 |
| HDL baseline | 0.362 | 0.822 | 0.023 | 0.66 | -1.26 to 1.98 |
| IHD | -0.552 | 0.764 | -0.038 | 0.471 | -2.06 to 0.95 |
| Sulfonylurea | -0.676 | 0.54 | -0.066 | 0.212 | -1.74 to 0.39 |
| Biguanide | 0.164 | 1.742 | 0.005 | 0.925 | -3.26 to 3.59 |
| Thiazolidinedione | 0.737 | 2.055 | 0.019 | 0.72 | -3.3 to 4.78 |
| SGLT2 | 0.575 | 0.479 | 0.063 | 0.23 | -0.37 to 1.52 |
| DPP4 | -0.633 | 1.742 | -0.019 | 0.717 | -4.06 to 2.79 |
| ~~Basal bolus Insulin~~ | ~~0.824~~ | ~~0.477~~ | ~~0.091~~ | ~~0.085~~ | ~~-0.11 to 1.76~~ |
| ~~Basal only~~ | ~~1.408~~ | ~~1.165~~ | ~~0.063~~ | ~~0.227~~ | ~~-0.88 to 3.7~~ |
| ~~Bolus only~~ | ~~0.21~~ | ~~0.757~~ | ~~0.015~~ | ~~0.781~~ | ~~-1.28 to 1.7~~ |
| ~~1mg vs. 0.5mg~~ | ~~-0.109~~ | ~~0.528~~ | ~~-0.011~~ | ~~0.837~~ | ~~-1.15 to 0.93~~ |
| Any Insulin | 1.278 | 0.502 | 0.133 | 0.011 | 0.29 to 2.27 |
